# Activity-regulated growth of motoneurons at the neuromuscular junction is mediated by NADPH oxidases

**DOI:** 10.3389/fncel.2022.1106593

**Published:** 2023-01-13

**Authors:** Daniel Sobrido-Cameán, Matthew C. W. Oswald, David M. D. Bailey, Amrita Mukherjee, Matthias Landgraf

**Affiliations:** Department of Zoology, University of Cambridge, Cambridge, United Kingdom

**Keywords:** motoneuron, plasticity, Drosophila, reactive oxygen species, NADPH oxidase, dual oxidase, Nox, aquaporin genes

## Abstract

Neurons respond to changes in the levels of activity they experience in a variety of ways, including structural changes at pre- and postsynaptic terminals. An essential plasticity signal required for such activity-regulated structural adjustments are reactive oxygen species (ROS). To identify sources of activity-regulated ROS required for structural plasticity *in vivo* we used the Drosophila larval neuromuscular junction as a highly tractable experimental model system. For adjustments of presynaptic motor terminals, we found a requirement for both NADPH oxidases, Nox and dual oxidase (Duox), that are encoded in the Drosophila genome. This contrasts with the postsynaptic dendrites from which Nox is excluded. NADPH oxidases generate ROS to the extracellular space. Here, we show that two aquaporins, Bib and Drip, are necessary ROS conduits in the presynaptic motoneuron for activity regulated, NADPH oxidase dependent changes in presynaptic motoneuron terminal growth. Our data further suggest that different aspects of neuronal activity-regulated structural changes might be regulated by different ROS sources: changes in bouton number require both NADPH oxidases, while activity-regulated changes in the number of active zones might be modulated by other sources of ROS. Overall, our results show NADPH oxidases as important enzymes for mediating activity-regulated plasticity adjustments in neurons.

## 1. Introduction

Reactive oxygen species (ROS) have commonly been associated with detrimental processes such as oxidative stress, toxicity, aging, neurodegeneration, and cell death because increases in ROS levels seen with aging and neurodegenerative disorders, including Parkinson's (Spina and Cohen, 1989) and Alzheimer's disease (Martins et al., 1986). However, it is appreciated that ROS are not simply cytotoxic agents, but more generally function as signaling molecules in a multitude of processes, (Rhee, 1999; Sauer et al., 2001) including growth factor signaling (Suzukawa et al., 2000; Goldsmit et al., 2001; Kamata et al., 2005; Nitti et al., 2010), wound healing (Razzell et al., 2013), and in development (Milton et al., 2011; Oswald M. C. et al., 2018; Oswald M. et al., 2018; Dhawan et al., 2021 for a reviews see Owusu-Ansah and Banerjee, 2009; Massaad and Klann, 2011; Wilson and González-Billault, 2015; Terzi and Suter, 2020).

During nervous system development, ROS signaling is involved at all stages, from neurogenesis to pathfinding to synaptic transmission (Knapp and Klann, 2002; Kishida and Klann, 2007; Massaad and Klann, 2011; Wilson and González-Billault, 2015; Wilson et al., 2018; Terzi and Suter, 2020). When studying ROS signaling *in vivo*, challenges include the ability to disentangle cell autonomous from indirect or systemic effects; or to determine sources and types of ROS. Using the fruit fly, *Drosophila melanogaster*, as a highly tractable experimental model system, genetic manipulations targeted to single motoneurons were able to identify hydrogen peroxide as a synaptic plasticity signal, generated as a consequence of neuronal overactivation and both necessary and sufficient for activity-regulated adaptive changes of synaptic terminal structure and transmission (Oswald M. C. et al., 2018; Dhawan et al., 2021). We found mitochondria to be a major source of activity-regulated hydrogen peroxide with opposing effects on the growth of pre- vs. postsynaptic terminals: at the presynaptic terminal of the neuromuscular junction (NMJ) overactivation and hydrogen peroxide cause increases in terminals (Milton et al., 2011; Oswald M. C. et al., 2018). This change in presynaptic terminal growth is mediated by activation of the JNK signaling pathway (Milton et al., 2011), and it utilizes the conserved Parkinson's disease-linked protein, DJ-1β, as a redox sensor, which regulates the PTEN-PI3 Kinase growth pathway (Oswald M. C. et al., 2018). In contrast, the size of postsynaptic dendritic arbors is negatively regulated by over-activation and activity-regulated hydrogen peroxide (Tripodi et al., 2008; Oswald M. C. et al., 2018; Dhawan et al., 2021). These studies using the Drosophila larval neuromuscular model system contrast with findings from cultured hippocampal neurons, which posit mitochondrially generated superoxide as the principal ROS signal downstream of over-activation (Hongpaisan et al., 2003, 2004). The extent to which both types of ROS operate as neuronal plasticity signals downstream of over-activation remains to be resolved, though it is possible that apparent discrepancies might be due to the use of different cellular models and/or a reflection of the degree of overactivation.

Another principal source of ROS are NADPH oxidases, whose location in the plasma membrane could facilitate sub-cellular signaling discrete from mitochondrial ROS production. NADPH oxidases are integral membrane proteins that mediate a single electron transfer from NADPH to oxygen, thereby converting it to superoxide (Lambeth, 2002). These enzymes are prevalent throughout the evolutionary ladder from Amoebozoa and fungi to higher plants and mammals. NADPH oxidases are involved in growth and plasticity during nervous system development (Serrano et al., 2003; Tejada-Simon et al., 2005; Kishida et al., 2006; Munnamalai and Suter, 2009; Munnamalai et al., 2014; Olguín-Albuerne and Morán, 2015; Wilson et al., 2015, 2016; Terzi and Suter, 2020). In contrast to mammalian genomes, which encode seven Nox isoforms (Nox 1–5 and Duox 1 and 2; Lambeth, 2002; Kawahara et al., 2007), *Drosophila melanogaster* encodes just two NADPH oxidases: dual oxidase (Duox) and a Nox-5 homolog (Nox). Enzymatic activity of both is calcium-regulated, *via* their N-terminal calcium binding EF-hands (Ha et al., 2005a,b, 2009; Moreira et al., 2010; Razzell et al., 2013). Curiously, the mouse genome does not encode a calcium-regulated Nox-5 homolog, which has therefore not been studied extensively *in vivo* (Kawahara et al., 2007). Recently, we identified the NADPH oxidase Duox as necessary in motoneurons to reduce their dendritic arbors in response to neuronal over-activation, an adaptive response to reduce the numbers of presynaptic inputs and thus synaptic drive (Zwart et al., 2013; Dhawan et al., 2021). We further found that these activity-regulated ROS, generated by Duox at the extracellular face of the plasma membrane, required the aquaporins, Bib and Drip; presumably for efficient entry into the cytoplasm to regulate dendritic growth and/or stability (Dhawan et al., 2021).

Here, we investigated the role of NADPH oxidases at the presynaptic terminal of the NMJ, whose growth response to neuronal over-activation is distinct to that of the dendritic compartment of the motoneuron. We show that the NADPH oxidases Duox and Nox are sources of activity-regulated ROS that mediate activity-regulated growth of NMJ terminals. In contrast to motoneuron dendrites, both NADPH oxidases function at the presynaptic NMJ, necessary and sufficient to elicit changes in growth. At the NMJ too, we find the aquaporins, Bib and Drip, are necessary for ROS signaling at the NMJ. This arrangement at the presynaptic NMJ terminal contrasts with their dendritic function within these motoneurons, where only Duox, but not Nox, is required. This differential requirement of Nox mirrors its sub-cellular localization, with Nox largely excluded from dendrites. Furthermore, at the postsynaptic compartment extracellular ROS, including from other neurons in the vicinity, act as local plasticity signals that cause reductions in dendritic arbor size (Dhawan et al., 2021).

## 2. Results

### 2.1. NADPH oxidases, Duox and Nox, are both required for activity-regulated growth at the neuromuscular junction

Mitochondria are a major source of activity-generated ROS, notably within the cytoplasm. Here, we sought to investigate the role of membrane localized ROS generators, the NADPH oxidases Nox and Duox, during activity-regulated adjustment of presynaptic terminals. As a highly tractable experimental model we used the well-characterized neuromuscular junction (NMJ) of the Drosophila larva (Frank et al., 2013). Specifically, we focused on the NMJ of the so called “anterior Corner Cell” (aCC), which innervates the most dorsal body wall muscle, known as muscle 1 (Crossley, 1978) or dorsal acute muscle 1 (DA1; Sink and Whitington, 1991; Bate, 1993; Landgraf et al., 1997; Baines et al., 1999, 2001; Hoang and Chiba, 2001; Choi et al., 2004). For cell-specific over-activation of aCC motoneurons, we used the established paradigm of targeted mis-expression of the warmth-gated cation channel, dTRPA1 Gain-of-Function (GoF; Hamada et al., 2008; Oswald M. C. et al., 2018; Dhawan et al., 2021). This allows aCC motoneurons to be selectively overactivated simply by placing larvae at >24°C, the temperature threshold for dTRPA1 ion channel opening (Pulver et al., 2009).

**Figure 1:**
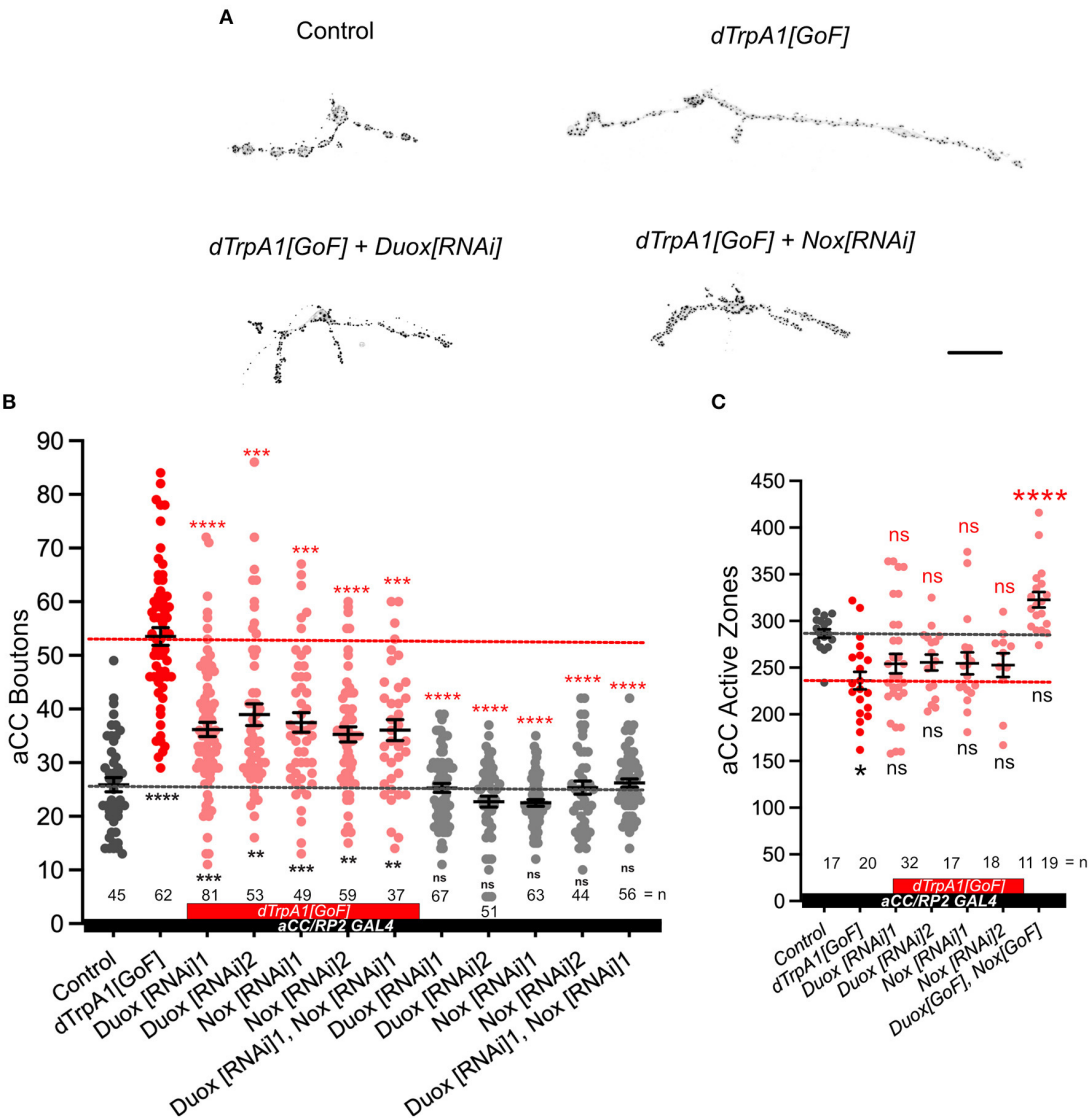
NADPH oxidases, dDuox and dNox, are both required for activity-regulated growth of the neuromuscular junction. **(A)** Representative images of aCC motoneuron terminals on their target muscle, DA1 (muscle 1, according to Crossley, 1978) in third instar larvae [100 h after larva hatching (ALH)] labeled with a *20xUAS-6xmCherry* reporter expressed by *aCC/RP2-Gal4* (see Section 4) and active zones (black spots): control (*aCC/RP2-Gal4/*+); dTrpA1 overactivated; dTrpA1 overactivated while either Duox or Nox is concomitantly knocked down *via* targeted RNAi [“*dTrpA1[GoF]* + *Duox[RNAi]*” and “*dTrpA1[GoF]* + *Nox[RNAi]*”]. **(B)** Dot-plot quantification shows NMJ bouton number increases in response to cell-specific activity increases. This phenotype is rescued by simultaneous NADPH oxidase knockdown. **(C)** Dot-plot quantification shows active zone number decreases following overactivation [*dTrpA1[GoF]*], but there are not significant differences when manipulating NADPH oxidases. Mean ± SEM, ANOVA, *p < 0.05, **p < 0.01, ***p < 0.001, and ****p < 0.0001. ns = not significant. Red asterisks indicate comparisons with the *dTrpA1[GoF]* group, while black indicate comparison with the un-manipulated wild type control. Scale bar = 20 μm.

First, we re-confirmed that at 25°C *dTrpA1[GoF]* in aCC motoneurons leads to significant increases in bouton number at the aCC-DA1 NMJ relative to non-manipulated controls, as previously shown (Oswald M. C. et al., 2018; Figure 1). An advantage of using cell-specific dTRPA1-mediated activity manipulations in this system is that these can be carried out at 25°C, a temperature considered optimal for *Drosophila melanogaster* development (Lachaise et al., 1988; Pool et al., 2012) and therefore generally considered neutral, while sufficient to mildly activate neurons that mis-express dTRPA1 (Pulver et al., 2009; Tsai et al., 2012).

Next, we tested the requirement for the two NADPH oxidases encoded in the Drosophila genome, Duox and Nox, in mediating these activity-regulated structural changes at the NMJ. To this end, we expressed RNAi transgenes for knocking down endogenous Duox or Nox in aCC motoneurons. By themselves, expression of *Duox[RNAi]* or *Nox[RNAi]* transgenes in aCC motoneurons have no measurable effect on NMJ morphology. However, in motoneurons that have been overactivated by *dTrpA1[GoF]*, the characteristic activity-induced bouton overgrowth phenotype is suppressed by co-expression of *Duox[RNAi]* or *Nox[RNAi]* transgenes, individually or combined (Figures 1A, B). Neuronal overactivation by *dTrpA1[GoF]* also causes a reduction in active zone numbers (Oswald M. C. et al., 2018). We find that simultaneous knockdown of NADPH oxidases seems to ameliorate active zone reductions, though differences were subtle and not statistically significant (Figure 1C). These results show that the membrane localized ROS generators, Nox and Duox, are required primarily for activity-regulated changes in presynaptic terminal growth, while the impact on presynaptic release sites was less clear.

### 2.2. Duox and Nox activity is sufficient for mediating structural changes at the NMJ

**Figure 2:**
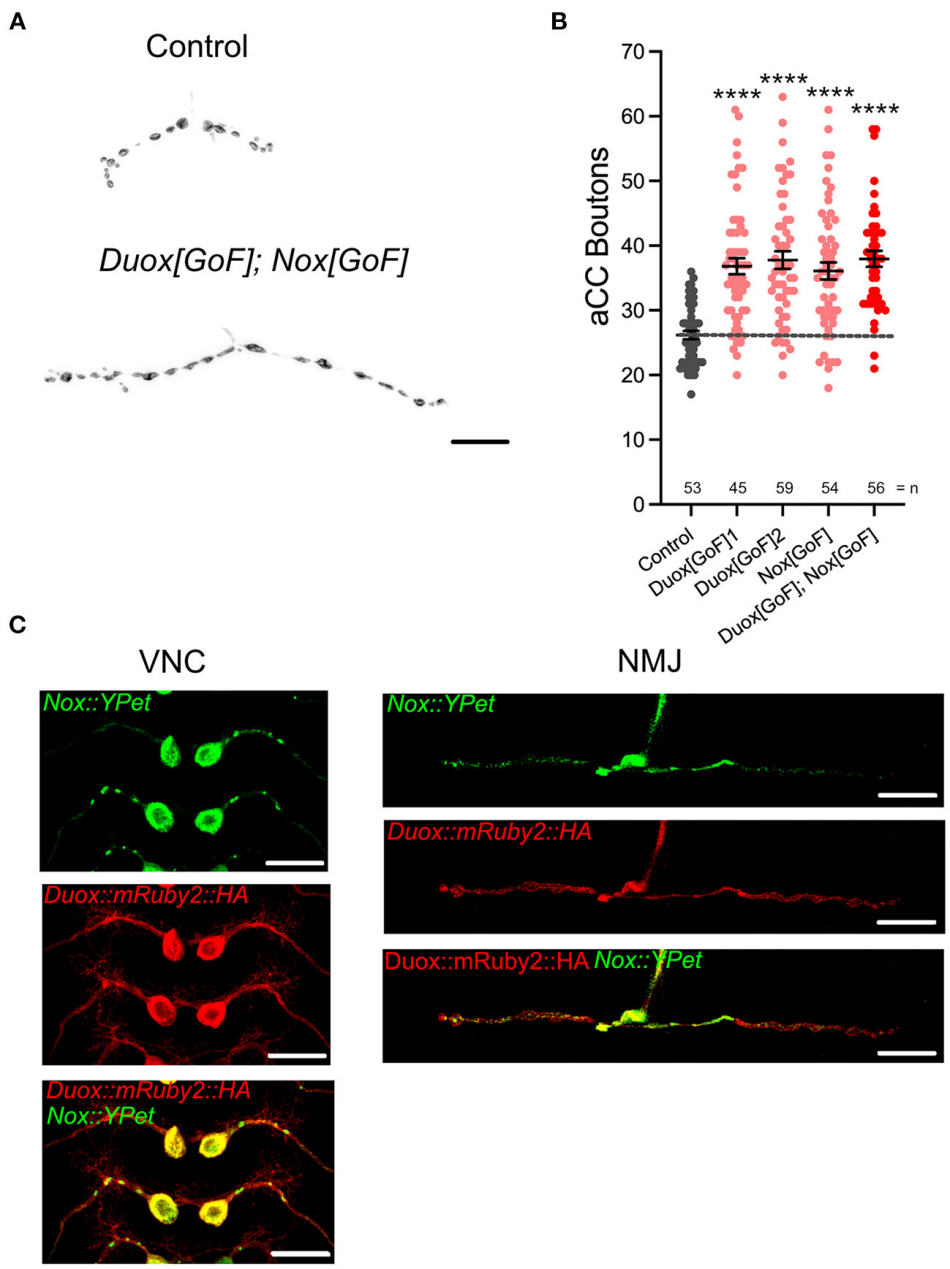
dDuox or dNox activity is sufficient for mediating structural changes at the NMJ. **(A)** Representative images of aCC presynaptic terminals on muscle DA1 from third instar larvae (100 h ALH) of control (*aCC/RP2-Gal4/*+) and those overexpressing *Duox[GoF]* and *Nox[GoF]*. **(B)** Dot-plot quantification shows NMJ bouton number increases in response to cell-specific over-expression of NADPH oxidases. **(C)** Localization of tagged Duox and Nox transgenes in neurons: representative confocal micrograph images of aCC somata and dendrites in the ventral nerve cord (VNC) and aCC presynaptic terminals at the DA1 muscle in third instar larvae (72 h ALH), showing subcellular localization of tagged over-expressed Duox::mRuby2::HA (in red) and Nox::YPet (in green). Mean ± SEM, ANOVA, ****p < 0.0001. Comparisons are made with the control group. Scale bar = 20 μm.

We next asked if the activity of these NADPH oxidases might also be sufficient for regulating presynaptic terminal growth. To test this, we induced a NADPH oxidase gain-of-function by overexpression of *Duox[GoF]* or *Nox[GoF]* transgenes in aCC motoneurons. Quantification showed comparable increases in bouton number at the NMJ as a consequence of overexpression of either *Duox[GoF]* or *Nox[GoF]*. No enhancement of this phenotype occurs when both are co-expressed (Figure 2). In contrast, active zone numbers are not significantly impacted by overexpression of either NADPH oxidase (Figure 1).

For the postsynaptic compartment, namely the dendritic arbor of motoneurons, we had previously shown that only Duox, but not Nox, has a role in activity-regulated plasticity (Dhawan et al., 2021). To further explore this difference in NADPH oxidase requirement between pre- vs. postsynaptic compartments, we generated tagged transgenes of both NADPH oxidases, UAS-Duox::mRuby2::HA and UAS-Nox::YPet. When expressed in aCC motoneurons to reveal subcellular localization, we see exclusion of Nox::YPet from the postsynaptic dendrites, while Duox::mRuby2::HA is fairly homogeneously distributed within the plasma membrane (Figure 2C). These patterns of distinct subcellular distributions, notably exclusion of Nox::YPet from dendrites, are compatible with the genetic manipulations phenotypes. They point to selective sorting of Nox to soma and presynaptic compartments, based on the fluorescently tagged transgene.

### 2.3. Aquaporin channel proteins Bib and Drip are necessary for NADPH oxidase-regulated structural changes at the NMJ

**Figure 3:**
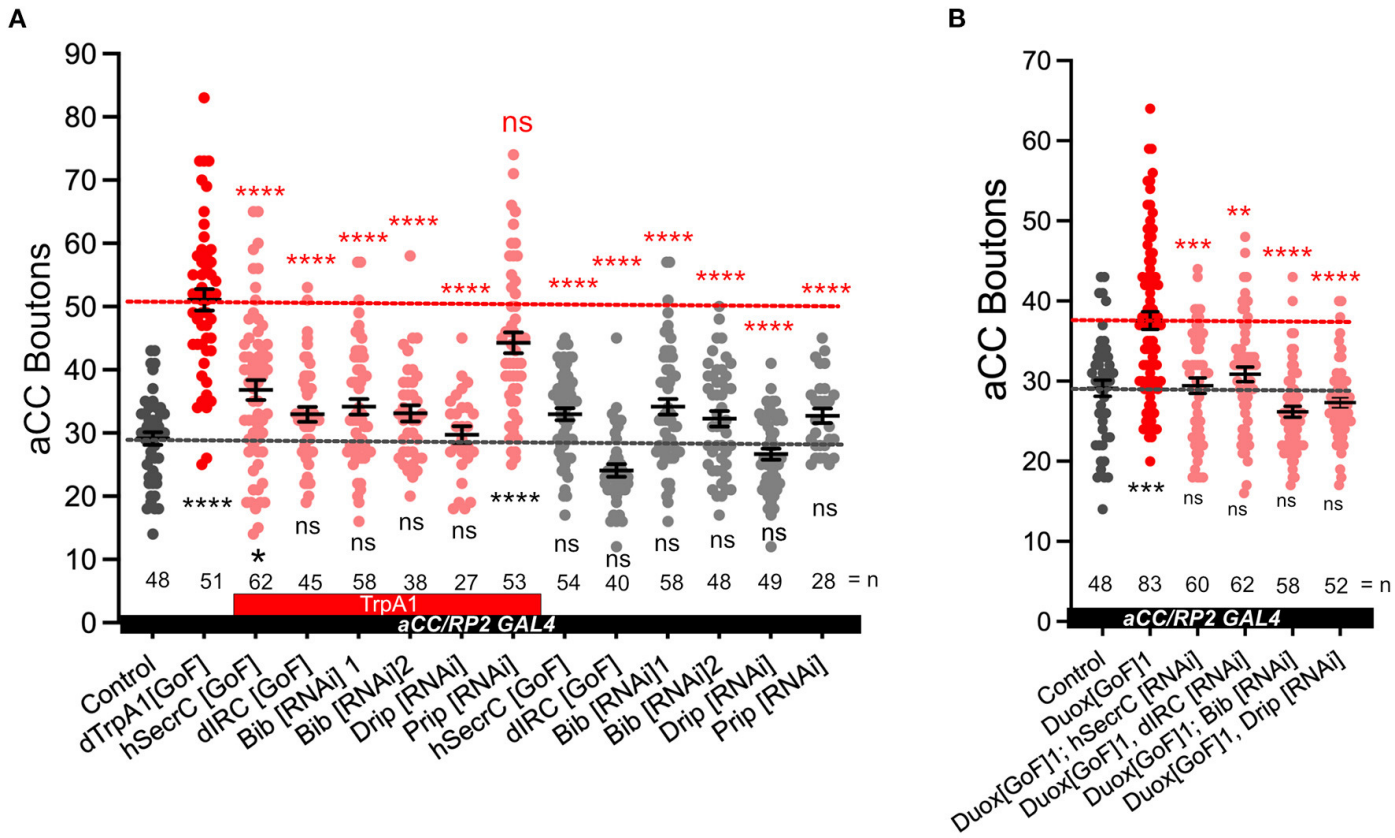
Aquaporins Bib and Drip are required for activity-regulated plasticity at the neuromuscular junction. **(A)** Dot-plot quantification shows NMJ bouton number increases in response to cell-specific activity and the rescue of the phenotype when secreted catalases are expressed *[GoF]* or aquaporins Bib or Drip are knocked down; control (*aCC/RP2-Gal4/*+). **(B)** Dot-plot quantification shows NMJ bouton number increases in response *Duox[GoF]* and the rescue of the phenotype when secreted catalases are expressed or aquaporins Bib or Drip are knocked down; control (*aCC/RP2-Gal4/*+). Mean ± SEM, Kruskal-Wallis test, *p < 0.05, **p < 0.01, ***p < 0.001, and ****p < 0.0001. ns = not significant. Red asterisks indicate statistical comparisons with the *dTrpA1[GoF]* group, while black asterisks comparison with the un-manipulated wild type control.

The NADPH oxidases Duox and Nox are transmembrane proteins that generate ROS at the extracellular face of the plasma membrane (Lambeth, 2002; Panday et al., 2015). We reasoned that if NADPH oxidase-generated ROS are indeed instrumental in activity-regulated adjustment of synaptic terminals, then neutralization of extracellular ROS should rescue NMJ phenotypes associated with NADPH oxidase overexpression. To test this, we mis-expressed in aCC motoneurons two different forms of catalases that are secreted to the extracellular space; a human version and the Drosophila immune-regulated catalase (IRC; Ha et al., 2005a,b; Fogarty et al., 2016). These catalases neutralize extracellular hydrogen peroxide by conversion to water. On their own, their mis-expression in aCC motoneurons has no significant impact on NMJ structure or size. To test the model of neuronal activity leading to NADPH oxidase activation, leading to extracellular ROS production, we co-expressed secreted catalase in aCC motoneurons while over-activating these with *dTrpA1[GoF]*. The presence of a secreted catalase suppresses the NMJ growth that would otherwise ensue with neuronal overactivation (Figure 3A). Similarly, NMJ over-growth stimulated by over-expression of Duox (*Duox[GoF]*) is also neutralized by co-expression of secreted catalase in the same neuron (Figure 3B). These experiments demonstrate that it is the presence of extracellular ROS, notably hydrogen peroxide generated by NADPH oxidases, which leads to activity-induced changes in NMJ growth.

Because NAPDH oxidases generate ROS extracellularly, we wanted to explore how extracellular ROS might enter the cell so as to act on intracellular signaling pathways that would regulate NMJ growth. Several studies, including one from this lab, have postulated a role for aquaporin channels, specifically those encoded by the genes Bib and Drip (Albertini and Bianchi, 2010; Dhawan et al., 2021; Dutta and Das, 2022). Indeed, for the presynaptic NMJ, we found that co-expression of *UAS-RNAi* constructs designed to knock down Bib or Drip, but not those for Prip, rescue NMJ growth phenotypes caused by dTRPA1-mediated overactivation. Expression of the *UAS-RNAi* constructs alone had no significant effect (Figure 3A). To further test the model that extracellular ROS generated by NADPH oxidases cause structural change at the NMJ, we overexpressed *Duox[GoF]* in aCC motoneurons and at the same time co-expressed *UAS-RNAi* constructs designed to knock down the aquaporin channel proteins *Bib[RNAi]* or *Drip[RNAi]*. In those neurons the *Duox[GoF]* NMJ growth phenotype is fully rescued (Figure 3B).

**Figure 4:**
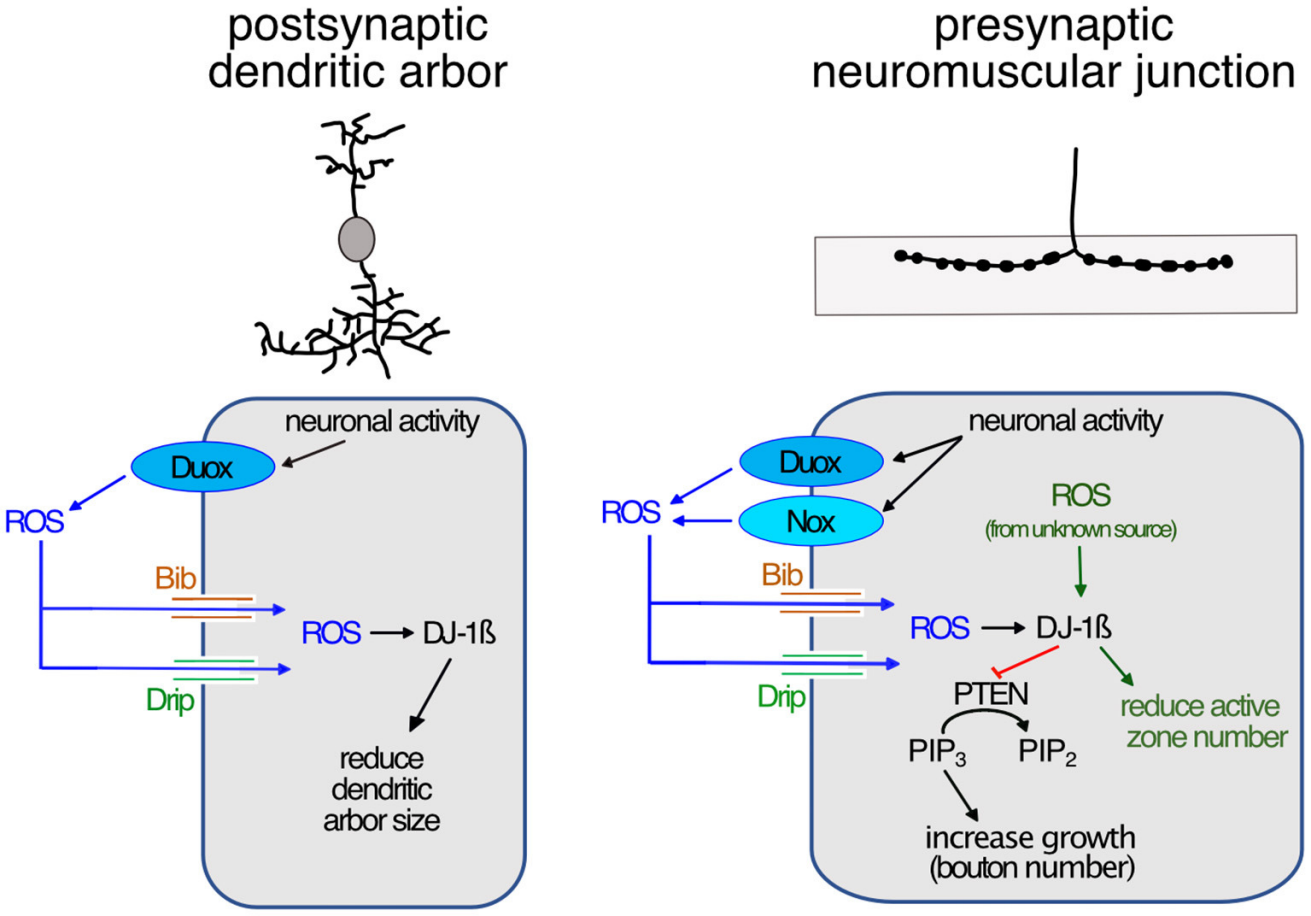
Model of activity-regulated plasticity at the postsynaptic dendritic arbor and the presynaptic neuromuscular junction mediated by ROS signaling. Neuronal activity leads to activation of calcium-regulated NADPH oxidases in synaptic terminals; of Duox only in postsynaptic dendrites, while at the presynaptic neuromuscular junction both Duox and Nox are activated. Extracellularly generated ROS reintroduced into the cytoplasm *via* aquaporin channels, including Bib and Drip. This leads to reduced dendritic growth, while at the presynaptic neuromuscular junction ROS promotes growth by modulating PI3Kinase signaling at the plasma membrane *via* oxidation of DJ-1ß. Oxidized DJ-1ß increases binding and inhibition of the PTEN phosphatase, thus causing increased PI3Kinase signaling activity, stimulating growth and addition of synaptic release sites. ROS from unknown sources regulate the active zone number.

In summary, our observations suggest that at the presynaptic NMJ, neuronal overactivation leads to activation of both NADPH oxidases, Duox and Nox, at the plasma membrane. These enzymes generate ROS at the extracellular face, which are then brought into the cytoplasm by aquaporin channels comprising Bib and Drip. Inside the cell, the ROS act on intracellular membrane-localized signaling pathways that regulate synaptic terminal structure and size, including the phosphatase PTEN and DJ-1ß, as previously shown (Oswald M. C. et al., 2018; Figure 4).

## 3. Discussion

ROS have increasingly been recognized as signaling molecules required for nervous system development and function, from regulating the dynamics of the growth cone cytoskeleton to synaptic transmission and learning (see Terzi and Suter, 2020). At the Drosophila NMJ, ROS have been shown necessary for activity-induced synaptic terminal growth (Oswald M. C. et al., 2018). ROS have also been shown causative and sufficient to induce changes at synaptic terminals; when accumulating as a result of physiological dysfunction, leading to oxidative stress (Milton et al., 2011), or following manipulations that increase ROS levels (Milton et al., 2011; Hussain et al., 2018; Peng et al., 2019). While mitochondria are a major source of cellular ROS (Murphy, 2009; Zorov et al., 2014; Sanz, 2016), it has remained unclear to what extent mitochondrial or indeed other sources of ROS, as explored in this study, directly impact effectors that regulate synaptic terminal growth. At the larval NMJ, different signaling pathways feed into growth regulation, notably Wnt (Budnik and Salinas, 2011; Koles and Budnik, 2012), BMP (Bayat et al., 2011; Berke et al., 2013; Sulkowski et al., 2014; Osses and Henríquez, 2015), PKA, CREB, and the transcription factor AP-1 (Davis et al., 1996, 1998; Sanyal et al., 2002, 2003; Davis, 2006; Walker et al., 2013; Cho et al., 2015; Davis and Müller, 2015). ROS might serve a general role in amplifying signaling pathway activity by disinhibition of pathway-associated protein kinases. For example, at the Drosophila NMJ, we previously showed that activity-regulated ROS inhibit the phosphatase PTEN, and thus disinhibit PI3Kinase, whose activity promotes synaptic terminal growth (Martín-Peña et al., 2006; Acebes and Morales, 2012; Jordán-Álvarez et al., 2012; Oswald M. C. et al., 2018). ROS have further been shown to modulate BMP signaling in sympathetic neurons (Chandrasekaran et al., 2015; Sánchez-de-Diego et al., 2019) and Wnt pathways in non-neuronal cells (Funato et al., 2006; Love et al., 2013; Rharass et al., 2014). The link between PKA and ROS is not well-understood though ROS can activate PKA in Amygdala neurons, increasing their excitability (Li et al., 2011). Changes in neuronal excitability can also be directly modulated by oxidation of any number of ion channels, for example of the K-channel auxiliary subunit, Hyperkinetic (Fogle et al., 2015; Kempf et al., 2019). We therefore hypothesize that ROS may provide neuronal activity-regulated modulation of multiple canonical synaptic plasticity pathways. In such a scenario, one might expect that the necessary spatial precision is achieved through distinct sources of ROS generation.

### 3.1. Differential requirements for NADPH oxidases in pre- vs. postsynaptic compartments

In this study, we focused on NADPH oxidases as generators of ROS that are ideally positioned to influence signaling at the plasma membrane. Working with the NMJ in the Drosophila larva as an experimental *in vivo* model system, we demonstrated that both NADPH oxidases, Nox and Duox, are required for activity-induced growth (Figure 1). Both enzymes are endowed with N-terminal calcium binding EF-hand motifs, linking their activity to intracellular calcium levels, as shown for Drosophila Duox (Ha et al., 2009; Rigutto et al., 2009; Razzell et al., 2013) and the vertebrate homolog, Nox5 (Bánfi et al., 2004; Millana Fañanás et al., 2020). Conversely, overexpression of either enzyme is sufficient to phenocopy such presynaptic terminal growth (Figure 2). Curiously, the requirement for NADPH oxidases in regulating dendritic growth is different, with only Duox, but not Nox, mediating activity-induced reduction of dendritic arbor size (Dhawan et al., 2021). This difference in pre- vs. postsynaptic compartment regulation is mirrored by their differential sub-cellular localization, with tagged Nox protein being effectively excluded from the postsynaptic dendritic arbors, unlike Duox (Figure 2C). Apart from this differential pre- vs. postsynaptic requirement, it is unclear to what extent Nox and Duox might perform different functions during activity-regulated growth. At the NMJ, where both are present and required, we found no difference in phenotypes following RNAi knockdown or mis-expression. Curiously, phenotypes were also comparable regardless of whether the expression of both enzymes was manipulated simultaneously or individually, suggesting either a saturation of phenotype or, speculatively, that Nox and Duox might operate in the same signaling pathway with their activation contingent on one another.

### 3.2. NADPH oxidases generate extracellular ROS and can mediate autocrine signaling

Because Nox and Duox generate ROS at the extracellular face they have the potential for inter-cellular signaling, as during wound healing (Niethammer et al., 2009; Razzell et al., 2013; Niethammer, 2016). Indeed, within the dense meshwork of neuronal processes and synapses of the CNS, we recently found that reduction of extracellular hydrogen peroxide in the vicinity of dendritic processes (by mis-expression of a secreted catalase) or attenuation of ROS entry into those dendrites (by knock-down of aquaporins), both cause significant dendritic over-growth (Dhawan et al., 2021). This suggests that within the densely innervated central neuropile, extracellular ROS generated, including by activity-regulated NADPH oxidases, might function as local signals to which neurons respond with adjustments of their synaptic terminals. This contrasts with the peripheral Drosophila larval NMJ, where we did not see any significant changes in synaptic terminal morphology following manipulations that would either reduce entry of ROS into the presynaptic terminal or reductions of extracellular ROS (Figure 3). These observations suggest that at the presynaptic NMJ, NADPH oxidases might be required primarily under conditions of elevated neuronal activity. While this suggests that at the presynaptic NMJ, NADPH oxidase-generated ROS might be engaged in autocrine signaling, there is potential for inter-cellular signaling with adjacent muscles and glia.

Extracellular ROS signaling at both pre- and postsynaptic compartments is underlined by the requirement for the aquaporin channel proteins, Bib and Drip (Figure 3; Dhawan et al., 2021). Some studies have questioned the extent to which Bib might function as an aquaporin, as unable to form effective water channels in a heterologous expression system (Tatsumi et al., 2009; Kourghi et al., 2018). However, in this and in a previous study (Dhawan et al., 2021), *Bib[RNAi]* knockdown produces synaptic terminal phenotypes indistinguishable from knockdown of *Drip[RNAi]*, or from mis-expression *[GoF]* of secreted forms of catalase (Dhawan et al., 2021). This suggests that Bib functions in the same pathway as the aquaporin Drip, potentially forming part of a heteromeric channel with permeability for hydrogen peroxide.

### 3.3. Independent, local regulation of pre- and postsynaptic terminal growth

Overactivation of neurons results in changes to both pre- and postsynaptic terminals, though it has been unclear in how far such changes in growth of input and output compartments might be co-ordinately regulated. Working with this experimental system we identified two sets of manipulations that suggest the growth of pre- and postsynaptic terminals can be regulated independently. First, in motoneurons that have been over-activated by mis-expression of *dTrpA1[GoF]*, RNAi knockdown of Nox has no effect on the activity-induced reduction of the postsynaptic dendrites, which receive all synaptic input from pre-motor interneurons (Nox protein appears to be excluded from dendrites); while at the presynaptic NMJ of those same neurons, activity-linked overgrowth is significantly suppressed by knockdown of *Nox[RNAi]*. This contrasts with the ability of *Duox[RNAi]* knockdown to suppresses *dTrpA1[GoF]* over-activation phenotypes at both pre- and postsynaptic terminals.

Second, RNAi knockdown alone of the genes coding for aquaporin channel proteins Bib or Drip cause significant dendritic overgrowth, without affecting the presynaptic NMJ. These manipulations suggest that, at least in Drosophila larval motoneurons, synaptic terminal growth can be regulated locally through ROS signaling, such that pre- and postsynaptic compartments can adjust independently from each other. This makes sense when viewing extracellular ROS as local signals for over-activation, to which cells respond by adjusting their synaptic terminals. It remains to be seen to what extent extracellular ROS might impact on the regulation of synaptic transmission.

In summary, it is increasingly appreciated that ROS are important signals, whose signaling capability is proportional to the spatiotemporal precision attained. Sub-cellular specificity of ROS generators, such as the NAPDH oxidases studied here, is an important facet.

## 4. Materials and methods

### 4.1. Fly genetics

*Drosophila melanogaster* strains were maintained on standard apple juice-based agar medium at 25°C. Fly strains used were: *OregonR* (#2376 Bloomington Drosophila Stock Center), *dTrpA1* in attP16 (Hamada et al., 2008; FBtp0089791), *Duox[RNAi]*1 (#32903 BDSC; FBtp0064955), *Duox[RNAi]*2 (#38916 BDSC; FBgn0283531), *Nox[RNAi]*1 (Ha et al., 2005a,b; FBal0191562), *Nox[RNAi]*2 (#32433 BDSC; FBgn0085428), *bib[RNAi]*1 (#57493 BDSC; FBtp0096443), *bib[RNAi]*2 (#27691 BDSC; FBtp0052515), *Drip[RNAi]*1 (#44661 BDSC; FBtp0090566), *Drip[RNAi]*2 (#106911 Vienna Drosophila Resource Center; FBtp0045814; Bergland et al., 2012), *Prip[RNAi]*2 (#44464 BDSC; FBtp0090258), *Duox[GoF]*1 (Ha et al., 2005a,b), *Duox::mRuby2::HA* (*Duox[GoF]*2; this paper), *Nox::YPet* (*Nox[GoF]*; this paper), *hSecrC[GoF] (human secreted catalase*; FBal0190351; Ha et al., 2005a,b; Fogarty et al., 2016), *dIRC[GoF] (Drosophila extracellular immune-regulated catalase*; FBal0191070, Ha et al., 2005a,b).

Transgene expression was carried out at 25°C targeted to RP2 and aCC motoneurons using the Gal4 expression line “*aCC/RP2-Gal4*”: *RN2-O-Gal4, UAS-FLP, tubulin84b-FRT-CD2-FRT-Gal4; RRFa-Gal4, 20xUAS-6XmCherry::HA* (Pignoni et al., 1997; Fujioka et al., 2003; Shearin et al., 2014). *RN2-GAL4* expression in RP2 and aCC motoneurons is restricted to the embryo, subsequently maintained by FLPase-gated *tubulin84B-FRT-CD2-FRT-GAL4* (Ou et al., 2008). We studied the mCherry expression to confirm that Gal4 expression is restricted to aCC and RP2 (Supplementary Figure 1). To study the localization of tagged *Nox::YPet* and *Duox::mRuby2::HA*, transgene expression was targeted to aCC motoneurons using *GMR94g006-Gal4* (#40701 BDSC; FBgn0053512; Pérez-Moreno and O'Kane, 2019). *pJFRC12-10XUAS-IVS-Nox-YPet* (GenBank OP716753) in VK00040 [cytogenetic 87B10] was generated by Klenow assembly cloning (tinyurl.com/4r99uv8m). Briefly, from pJFRC12-10XUAS-IVS-myr-GFP plasmid DNA we removed the coding sequence for *myr::GFP* using XhoI and XbaI, and replaced it with *Nox* cDNA from DGRC clone FI15205 (kindly provided by Kenneth H. Wan, DGRC Stock 1661239; https://dgrc.bio.indiana.edu//stock/1661239; RRID:DGRC_1661239), its 3' stop codon replaced by a flexible glycine-serine linker, followed by YPet (Nguyen and Daugherty, 2005). Similarly, we created *pJFRC12-10XUAS-IVS-Duox-mRuby2-HA* (GenBank OP716752) in landing site VK00022 [cytogenetic 57A5] using *Duox* cDNA kindly provided by Won-Jae Lee, its 3' stop codon replaced by a flexible glycine-serine linker, followed by mRuby2 (Lam et al., 2012), followed by another glycine-serine flexible linker and four tandem repeats of the hemagglutinin (HA) epitope. Transgenics were generated *via* phiC31 integrase-mediated recombination (Bischof et al., 2007) into defined landing sites by the FlyORF Injection Service (Zürich, Switzerland).

### 4.2. Dissections and immunocytochemistry

Flies were allowed to lay eggs on apple-juice agar based medium overnight at 25°C. Larvae were reared at 25°C on yeast paste, avoiding over-crowding. Precise staging of the late wandering third instar stage was achieved by: (a) checking that a proportion of animals from the same time-restricted egg lay had initiated pupariation; (b) larvae had reached a certain size and (c) showed gut-clearance of food [yeast paste supplemented with Bromophenol Blue Sodium Salt (Sigma-Aldrich)]. Larvae were dissected in Sorensen's saline, fixed for 5 min at room temperature in Bouins fixative or 10 min 4% paraformaldehyde (Agar Scientific) when staining for GFP/YPet epitopes, as detailed (Oswald M. C. et al., 2018). Wash solution was Sorensen's saline containing 0.3% Triton X-100 (Sigma-Aldrich) and 0.25% BSA (Sigma-Aldrich). Primary antibodies, incubated overnight at 10°C, were: Goat-anti-HRP Alexa Fluor 488 (1:1000; Jackson ImmunoResearch Cat. No. 123-545-021), Rabbit-anti-dsRed (1:1000; ClonTech Cat. No. 632496), Mouse nc82 (Bruchpilot; Developmental Studies Hybridoma Bank Cat No nc82), Chicken anti-GFP (1:5000; abcam Cat No ab13970); secondary antibodies, 2 h at room temperature: Donkey anti-mouse Alexa Fluor 647; Donkey-anti-Rabbit CF568 (1:1200; Biotium Cat. No. 20098), Donkey anti-Chicken CF488 (1:1000; Cambridge Bioscience Cat No 20166), and goat anti-Rabbit Atto594 (1:1000; Sigma-Aldrich Cat No 77671-1ML-F). Specimens were cleared in 70% glycerol, overnight at 4°C, then mounted in Mowiol.

Each experiment was performed at least two independent times. The “control” genotype is *aCC/RP2-Gal4/*+ generated by crossing wild type Oregon R flies to the *aCC/RP2-Gal4* line.

### 4.3. Image acquisition and analysis

Specimens were imaged using a Leica SP5 point-scanning confocal, and a 63x/1.3 N.A. (Leica) glycerol immersion objective lens and Leica Application Suite Advanced Fluorescence software. Confocal images were processed using ImageJ (to quantify active zones) and Affinity Photo (Adobe; to prepare figures). Bouton number of the NMJ on muscle DA1 from segments A3-A5 was determined by counting every distinct spherical varicosity along the NMJ branch.

To study if genetic manipulations targeted to aCC and RP2 motoneurons change muscle size we measured surface area of DA1 muscles, imaged with DIC optics using a Zeiss Axiophot microscope and a Plan-Neofluar 10x/0.3 N.A. objective lens. Images were taken with an Orca CCD camera (Hamamatsu) and muscle surface area was determined using ImageJ by multiplying muscle length by width. Differences in animal or muscle growth would lead to clear correlations between muscle surface area and bouton number. No changes in animal growth were observed, irrespective of aCC manipulation. In line with this, quantification of key representative experiments, covering most transgenic lines and conditions where genetic manipulation of aCC motoneurons cause significant changes in bouton number, shows no statistically significant differences in average muscle size. Correlating individual muscle size with bouton number shows that the biggest differences in muscle surface area are due to dissection artifact, e.g., extent of stretching larval filets (see Supplementary Figure 2). Taking account of this, bouton numbers are shown as raw counts, not normalized to average muscle surface area.

Representative schematics, drawings and plates of photomicrographs were generated with Affinity Photo (Serif Ltd., United Kingdom).

### 4.4. Statistical analysis

All data handling was performed using Prism software (GraphPad). NMJ bouton number data were tested for normal/Gaussian distribution using the D'Agostino-Pearson omnibus normality test. For normally distributed data one-way analysis of variance (ANOVA), with Tukey's multiple comparisons test was applied, while for non-normal distributions Kruskal-Wallis test was applied.

## Data availability statement

The datasets presented in this study can be found in online repositories. The names of the repository/repositories and accession number(s) can be found at: https://www.ncbi.nlm.nih.gov/genbank/, OP716752 and OP716753.

## Author contributions

DS-C, MO, AM, and ML conceived of the study and wrote the manuscript. DB cloned Duox and Nox transgenes. ML generated transgenic stocks. DS-C and MO carried out all experiments and analyzed data. All authors contributed to the article and approved the submitted version.

## Supplementary material

Supplementary Figure 1 aCC/RP2 Gal4. **(A)** Schematic representation of internal muscles of a 3rd instar larva with muscle DA1 shown in magenta. The highlighted area was imaged, shown in **(B)**; **(B)** Peripheral projections of Gal4-expressing aCC and RP2 motoneurons were visualized by UAS-6xmCherry. HRP staining reveals all neurons, the composite with mCherry shows specificity of Gal4 restricted to aCC and RP2 motoneurons. Scale bar: 100 μm.

Supplementary Figure 2 Muscles size. **(A)** Dot-plot quantification shows no statistically significant differences in average muscle surface area (MSA) between genotypes, including with mis-expression of UAS-dTrpA1 (red). Mean ± SEM, Kruskal-Wallis test. **(B)** Linear regression using the control data shows not correlation between aCC NMJ terminal bouton numbers and muscle size, *p*-value = 0.7866.
