# Supplementary figures and images for: Activity-regulated growth of motoneurons at the neuromuscular junction is mediated by NADPH oxidases
Source: Front Cell Neurosci. 2023 Jan 13;16:1106593. doi: 10.3389/fncel.2022.1106593 (PMC9880070; doi:10.3389/fncel.2022.1106593)

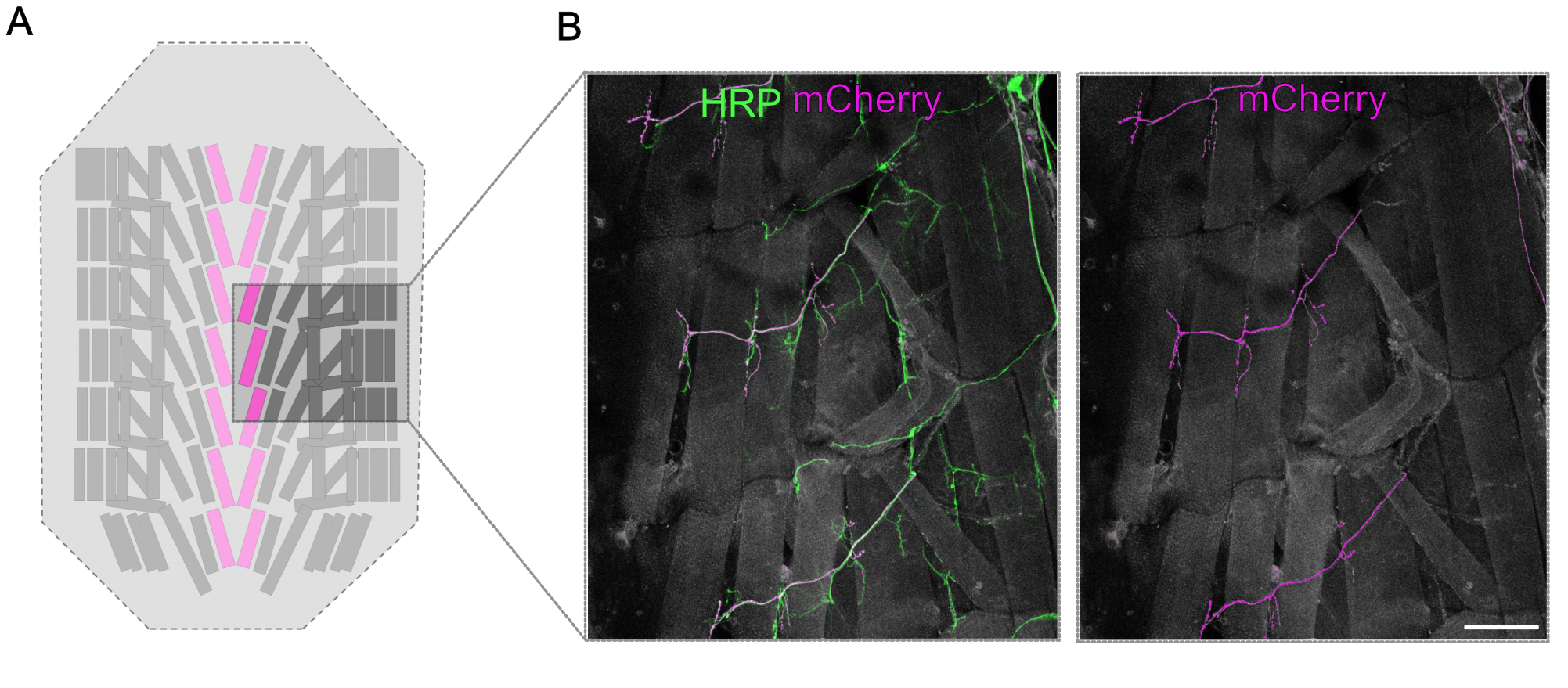

Supplement: Supplementary Figure 1 — aCC/RP2 Gal4. (A) Schematic representation of internal muscles of a 3rd instar larva with muscle DA1 shown in magenta. The highlighted area was imaged, shown in (B); (B) Peripheral projections of Gal4-expressing aCC and RP2 motoneurons were visualized by UAS-6xmCherry. HRP staining reveals all neurons, the composite with mCherry shows specificity of Gal4 restricted to aCC and RP2 motoneurons. Scale bar: 100 μm. [file Image_1.TIFF]

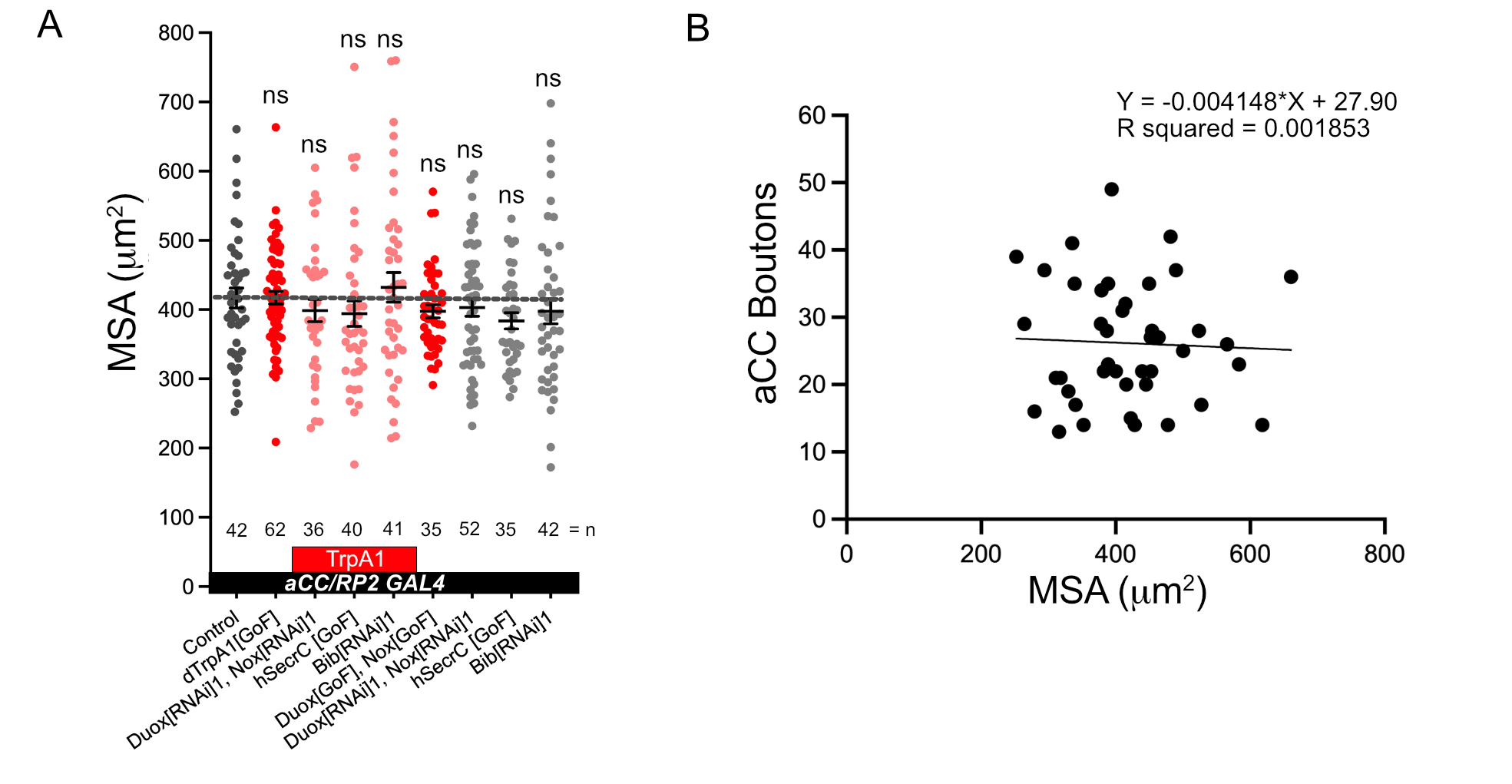

Supplement: Supplementary Figure 2 — Muscles size. (A) Dot-plot quantification shows no statistically significant differences in average muscle surface area (MSA) between genotypes, including with mis-expression of UAS-dTrpA1 (red). Mean ± SEM, Kruskal-Wallis test. (B) Linear regression using the control data shows not correlation between aCC NMJ terminal bouton numbers and muscle size, p-value = 0.7866. [file Image_2.TIFF]
